# miRNA-22 as a Candidate Diagnostic Biomarker for Coronary Slow Flow

**DOI:** 10.1155/2020/7490942

**Published:** 2020-07-26

**Authors:** Tong Chen, Zhen-Yu Wang, Chuan-Chang Li

**Affiliations:** ^1^Department of Geriatric Medicine, Xiangya Hospital, Central South University, Changsha 410008, China; ^2^Department of Cardiovascular Medicine, The Second Xiangya Hospital, Central South University, Changsha 410010, China

## Abstract

**Background:**

Coronary slow flow (CSF) refers to the phenomenon of delayed distal flow in the absence of lesions detected on coronary angiography. Although the detection rate of CSF has been increasing in clinical practice, early diagnosis is difficult and the factors contributing to this condition remain unclear. Given the increasing demonstration of the roles of microRNAs (miRNAs) in disease and as diagnostic biomarkers, the aim of this study was to analyze the expression of serum miRNA-22 in patients with CSF detected using coronary angiography and its diagnostic efficacy.

**Methods and Results:**

A retrospective analysis including 44 patients with CSF and 42 patients with normal coronary flow (control group) was conducted. Additionally, all included patients either did not have visually estimated coronary artery stenosis or had <50% stenosis. Plasma samples were collected from patients in these two groups, and the levels of miRNA-22 were detected. The receiver operating characteristic (ROC) curve was plotted to evaluate the diagnostic efficiency of serum miRNA-22 in the context of CSF.

**Results:**

The expression of serum miRNA-22 was significantly higher in the CSF patients than in the control subjects (*P* < 0.0001). The area under the ROC curve for miRNA-22 in diagnosing CSF was 0.8293 (95% confidence interval: 0.7313–0.9272), with a sensitivity of 75.0% and specificity of 88.1%.

**Conclusions:**

The expression of serum miRNA-22 in CSF is upregulated compared to that in subjects with normal coronary flow and shows relatively high clinical diagnostic efficiency, suggesting a new potential biomarker for the early diagnosis of CSF.

## 1. Introduction

In 1972, Tambe et al. first reported cases of patients with symptoms of chest pain accompanied by a significantly reduced blood flow rate in the absence of lesions detected on coronary angiographs [1]. This phenomenon is termed coronary slow flow (CSF), which refers to normal or near-normal major branches in the coronary arteries on coronary angiography but delayed and slow distal blood flow in vessel imaging [2–4]. CSF has been increasingly recognized with the popularization of coronary angiography, and it occurs in approximately 7% of patients with suspected coronary heart disease [5]. This trial demonstrated the high incidence of CSF, therefore warranting sufficient attention from clinicians. Despite the presence of lesions in blood vessels, the effective vascular lumen diameter does not decrease, which prevents the detection of vascular stenosis by coronary angiography. Consequently, early vascular lesions are not easily detected in patients with CSF by routine coronary angiography. Therefore, identification of markers that could enable the early diagnosis of CSF is clinically significant.

MicroRNAs (miRNAs) are endogenous noncoding small RNAs (22–23 nucleotides in length) that have recently been identified as having a wide range of functions with respect to regulating gene expression for influencing cell proliferation, differentiation, and migration [6–9]. The miRNA expression profile is altered in diseased tissues/organs, resulting in a disease-specific-expression pattern; therefore, miRNAs have emerged as important biomarkers for disease diagnosis [10–12]. However, the role of miRNAs in CSF is still not clear. Although the pathophysiological mechanism of CSF remains unclear, endothelial dysfunction is widely considered to play an important role [13–15]. Numerous studies have indicated that miRNA-22 plays a key role in regulating endothelial function [16, 17]. However, the associations among miRNA-22, endothelial function, and CSF have not been investigated until now. Therefore, in the present study, we evaluated the diagnostic value of miRNA-22 in the context of CSF among patients who underwent coronary angiography.

## 2. Materials and Methods

### 2.1. Study Subjects

We retrospectively analyzed the data of 1256 patients who underwent elective coronary angiography from March 2018 to March 2019 at the Department of Internal Medicine of the Xiangya Hospital. The inclusion criteria of this study were patients who either did not have visually estimated coronary artery stenosis or had <50% stenosis, which was defined as insignificant coronary artery stenosis, being consistent with many other studies [18, 19]. The following exclusion criteria were used: (1) acute coronary syndrome; (2) patients who underwent percutaneous coronary intervention or thrombolysis and patients with coronary artery ectasia or spasms; (3) other organic heart diseases such as cardiomyopathy, severe valvular heart disease, congenital heart disease, or severe cardiac insufficiency; (4) comorbid severe hepatic or renal impairment; (5) comorbid infection; (6) comorbid systemic inflammatory disorder; and (7) comorbid malignancy. The Judkins technique [20] was used for performing coronary angiography through the radial artery or femoral artery for all patients. The corrected thrombolysis in myocardial infarction (TIMI) frame count (CTFC) was used to evaluate the coronary artery blood flow rate using a video recording of the coronary artery, and a CTFC frame count >27 was used to make a diagnosis of CSF. Based on this criterion, 44 patients were diagnosed with CSF, including 32 men and 12 women. Meanwhile, 42 subjects with CTFC frame count <27 were chosen from the same cohort as controls, including 30 men and 12 women. All the 86 chosen patients were included in this study for comparison of the miRNA profiles. This retrospective clinical study was approved by the Ethics Committee of the Xiangya Hospital of Central South University (Changsha, Hunan Province, China).

### 2.2. Assessment of CTFC

All patients underwent coronary angiography with the standard Judkins method. Coronary flow rates were assessed by TFC, a classical method described by Gibson et al. [21]. Standard images were obtained at 30 frames per second. The first frame used for TIMI frame counting is the frame in which the dye fully enters both sides of the wall at the beginning of the coronary artery and moves forward smoothly. The final frame is recognized as the frame wherein the dye enters the anatomical landmark of the distal vessels. The anatomical landmarks are characterized as distal apical “eight-character” bifurcation in the left anterior descending (LAD), the furcation of the distal blunt margin branch in the left circumflex artery (LCX), and the first posterior branch of the left ventricle in the right coronary artery (RCA). Because the LAD is generally longer than the LCX and RCA, the TIMI of LAD was divided by 1.7 to obtain the CTFC. The mean TIMI frame count for each subject was obtained by adding the CTFC of the LAD to that of the LCX and RCA and then dividing the obtained value by 3 [21, 22].

### 2.3. Routine Blood Biochemical Tests

Before coronary angiography, 10 mL of venous blood was collected from all study subjects. The Hitachi 7600 fully automated biochemistry analyzer was used to measure the levels of fasting plasma glucose, total cholesterol, triglycerides, low-density lipoprotein cholesterol, and high-density lipoprotein cholesterol.

### 2.4. Real-Time Polymerase Chain Reaction (PCR) for miR-22 Detection

Five milliliters of whole venous blood was collected in ethylenediaminetetraacetic acid tubes and left to stand for 30 min at room temperature. The tubes were centrifuged at 4°C and 1200 ×g for 15 min and the serum in the upper layer was aspirated into 1.5 mL EP tubes. TRIzol (ThermoFisher Scientific, USA) was used for sample lysis and total RNA extraction before reverse transcription. Complementary DNA (cDNA) was synthesized under the following reaction conditions: 16°C for 15 min, 42°C for 60 min, 85°C for 5 min, and 4°C for 5 min. qPCR was performed using the SYBR Premix Ex Taq kit (Takara) on an ABI 7500 real-time PCR system (Applied Biosystems, USA). Proprietary qPCR primers were used, which were designed and validated by a commercial company (RiboBio, Guangzhou, China). qPCR was performed in triplicate for each sample. The 2^−ΔΔCt^ method was used to calculate the relative miRNA-22 expression in exosomes, in which ΔCt = Ct_miRNA-21_–Ct_U6_ and ΔΔCt = ΔCt_CSF group_–ΔCt_control group_, where Ct number is the number of amplification cycles for the fluorescence reaction intensity.

### 2.5. Statistical Analysis

SPSS 19.0 statistical software (SPSS Inc., Chicago, IL, USA) was used for data analysis. Quantitative data are presented as mean ± standard deviation. Student's *t*-test was employed for intergroup comparisons and one-way analysis of variance was employed for multiple group comparisons. Qualitative data are expressed as the number of subjects and percentage, and the chi-square test was employed for intergroup comparisons of these variables. The receiver operating characteristic (ROC) curve was used to evaluate the diagnostic performance of miRNA-22 in CSF. A difference with *P* < 0.05 was considered to be statistically significant.

## 3. Results

### 3.1. Basic Clinical Information

Table 1 shows the basic clinical data of the CSF and control groups. There were no statistically significant differences with respect to sex, age, body mass index, systolic blood pressure, diastolic blood pressure, heart rate, blood glucose, blood lipids, smoking status, alcohol consumption, or clinical drugs between the two groups (all *P* > 0.05).

### 3.2. TIMI Blood Flow Evaluation

As shown in Table 2, the TIMI blood flow frame counts in the LAD artery, LCX, and RCA, and the mean TIMI frame count were all significantly higher in the CSF group than in the control group (*P* < 0.001).

### 3.3. Expression of miRNA-22 in the CSF Group and Control Group

**Figure 1:**
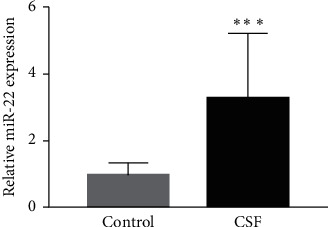
Validation of miRNA-22 expression in the plasma of CSF patients and controls by RT-qPCR (^*∗∗∗*^*P* < 0.001 vs. control group). RT-qPCR, reverse transcription quantitative polymerase chain reaction; CSF, coronary slow flow.

As shown in Figure 1, the expression of serum miRNA-22 was significantly upregulated in the CSF group relative to that in the control group.

### 3.4. Relationship between miRNA-22 and TIMI Grade

**Figure 2:**
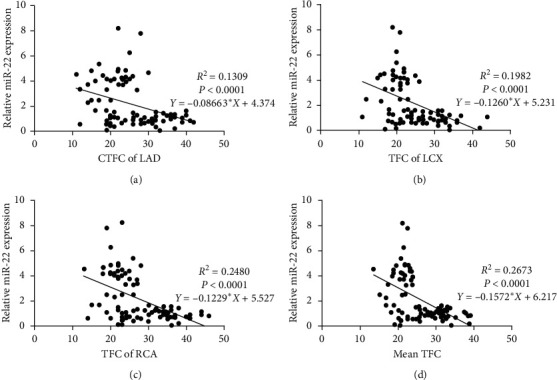
Correlation between miRNA-22 expression and four different CSF TIMI grades. Univariate linear regression analysis was used to evaluate the correlation between miRNA-22 expression and CTFC of LAD (a), TFC of LCX (b), TFC of RCA (c), and mean TFC (d) (*P* < 0.001 vs. four different CSF TIMI grades). CSF, coronary slow flow; TIMI, thrombolysis in myocardial infarction; CTFC, corrected TIMI frame count; LAD, left anterior descending artery; LCX, left circumflex artery; RCA, right coronary artery; mean TFC, mean TIMI frame count.

Univariate linear regression analysis showed that miRNA-22 expression was negatively correlated with the CSF TIMI grade (Figure 2).

### 3.5. Predictive Value of miRNA-22 for CSF Diagnosis

**Figure 3:**
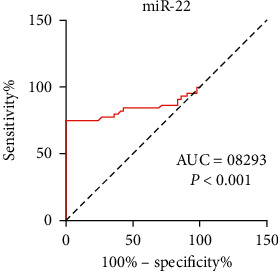
Diagnostic value of miR-22 for CSF. ROC curve analysis of miR-22. CSF, coronary slow flow; ROC, receiver operating characteristic.

The area under the ROC curve was 0.8293 (95% confidence interval: 0.7313–0.9272, *P* < 0.001) with 75.0% sensitivity and 88.1% specificity (Figure 3), suggesting that miRNA-22 has high accuracy for predicting CSF.

## 4. Discussion

To our knowledge, this is the first study to identify miRNA-22 as a potential early diagnostic biomarker of CSF. The expression of miRNA-22 was found to be upregulated in patients with CSF, and ROC analysis and univariate linear regression analysis of the TIMI frame count further indicated that miRNA-22 had significant diagnostic power for CSF.

CSF refers to a phenomenon whereby normal or near-normal major branches of the coronary arteries are observed on coronary angiography but there is delayed blood flow in distal blood vessel upon imaging [2–4]. There is now substantial evidence demonstrating that CSF is a main cause of clinical cardiovascular events such as resting and exertional angina and even myocardial infarction [2, 23–25], which severely affect quality of life. However, there is still no effective and appropriate method for the early diagnosis of CSF, which may result in prolonged pain and damage. Hence, further research on the pathogenesis, pathophysiological process, diagnosis, and treatment of CSF is essential, which can lead to identification of early CSF diagnostic markers.

miRNAs are highly conserved with the primary function of negatively regulating gene expression at the posttranscription level. miRNAs are broadly expressed in tissues and demonstrate high tissue specificity; approximately 60% of the human genes are regulated by miRNAs. miRNAs are also present in many types of body fluids such as plasma and serum, and extracellular circulating miRNAs are found enclosed within various vesicles, such as microvesicles, exosomes, and apoptosomes [26]. Circulating miRNAs are also highly stable and will not degrade if stored at room temperature for 24 h or repeatedly freeze-thawed. Moreover, miRNA expression is tissue-specific and responds rapidly to changes in the body. These characteristics suggest that miRNAs can be suitable biomarkers for the diagnosis and prognosis of diseases [27–29]. In recent years, numerous studies have found that miRNAs play an important role in the diagnosis and prognosis of cardiovascular diseases. Chen et al. [30] reported that miRNA-17-5p showed good sensitivity and specificity as a diagnostic marker for coronary heart disease, and circulating miRNA-499-5p levels were associated with risk of death in elderly patients after non-ST elevation myocardial infarction [31]. These studies suggested a crucial role of miRNAs in the occurrence and development of coronary atherosclerosis.

Although the pathophysiological mechanism of CSF remains unclear, several potential mechanisms have been suggested. Inflammation [32], vasoconstrictors [33], and even elevated levels of certain blood cells [34] may have direct and indirect effects on the process of CSF. Arksan et al. found that serum neutrophil gelatinase-associated lipocalin level was elevated in CSF patients, which indicated that inflammation, especially active inflammation, played a very important role in the CSF process, as neutrophil gelatinase-associated lipocalin would be released after active inflammation, such as that in acute organ injury [32]. Zengin et al. showed that serum urotensin-II levels were found to be significantly higher in the CSF group, suggesting that UII may be one of the factors involved in the pathogenesis of CSF [33]. Soylu et al. indicated that increases in hematocrit levels and eosinophil and basophil counts may have direct or indirect effects on the rate of coronary blood flow [34]. Based on these three previous studies, the pathophysiological mechanism of CSF is still unclear and it seems impossible to explain the CSF process by only one mechanism. However, among all the suggested underlying mechanisms, endothelial dysfunction is the most widely recognized [13–15]. Meanwhile, numerous studies have indicated that miRNA-22 plays a key role in regulating endothelial function. Tang et al. reported that the long noncoding RNA *MALAT1* protects the endothelium against oxidized low-density lipoprotein-induced dysfunction via upregulating the expression of the miRNA-22-3p target genes *CXCR2* and *AKT* [16]. Zheng et al. further showed that miRNA-22 induces endothelial progenitor cell senescence by targeting *AKT3* [17]. However, the associations among miRNA-22, endothelial function, and CSF have not been investigated until now. The present study suggests a potential role of miRNA-22 in CSF, warranting further detailed studies on the underlying mechanism, including the associated signaling pathways. In our study, we found that the serum miRNA-22 levels in CSF patients were significantly higher than those in the control group with normal coronary flow. Further evaluation based on the ROC curve showed high sensitivity and specificity, suggesting that miRNA-22 has good diagnostic performance with respect to CSF.

Huang et al. recently showed that the serum level of miRNA-155 was upregulated in patients with CSF compared with that of the control group, indicating that miRNA-155 could be another potential biomarker for CSF [35]. Although they enrolled more patients in their study (132) compared to the present study (86), we found a higher area under the ROC value (0.782 vs. 0.829) and a higher expression level of miRNA (0.023 vs. 3.28) despite using U6 as the reference gene in both studies. Hence, based on the evidence currently available, miRNA-22 may be a more promising and appropriate noninvasive biomarker compared with miRNA-155 for the early clinical diagnosis of CSF.

Although we detected a strong association between serum miRNA-22 levels and CSF, key limitations of the study need to be mentioned. First, only 86 patients were enrolled in this study, which is an insufficient number to make a substantial conclusion. Therefore, a large-scale study is required to substantiate our results and to further validate the specificity and sensitivity of the candidate biomarkers. Second, as a retrospective study, bias may arise from many factors. For example, though a common and useful method of selection and detection was applied in our study, selection bias or detection bias may lead to a spurious association between the predictor variable and outcome in the study sample, but not in the population, which is unavoidable. Finally, an experimental model should be established to further verify the targets of miRNA-22 and its role in the progression of CSF.

## 5. Conclusion

In summary, the relative expression of serum miRNA-22 is increased in patients with CSF, suggesting that changes in miRNA-22 expression may be involved in the occurrence of CSF. Serum miRNA-22 may serve as a biomarker for the early diagnosis of CSF. Further studies with a larger sample size are required to validate the association between serum miRNA levels and CSF to provide a reference for the clinical diagnosis and treatment of CSF.

## Figures and Tables

**Table 1:** General clinical data.

Characteristic	Control group (*n* = 42)	Coronary slow flow group (*n* = 44)	*P* value
Male (*n*, %)	30 (71.43)	32 (72.73)	0.8948
Age (years)	54.19 ± 6.213	56.48 ± 9.891	0.2052
BMI (kg/m^2^)	25.38 ± 2.083	25.57 ± 3.022	0.7400
Systolic pressure (mmHg)	125.9 ± 10.22	122.5 ± 7.981	0.0840
Diastolic blood pressure (mmHg)	73.93 ± 6.968	75.52 ± 7.013	0.2935
Heart rate (bpm)	72.40 ± 6.409	73.00 ± 7.314	0.6897
Fasting blood glucose (mmol/L)	5.363 ± 0.7273	5.641 ± 1.089	0.1691
Triglyceride (mmol/L)	1.522 ± 0.4450	1.626 ± 0.4848	0.3004
Total cholesterol (mmol/L)	4.620 ± 1.121	4.710 ± 1.252	0.7262
High-density lipoprotein (mmol/L)	1.177 ± 0.2796	1.156 ± 0.2024	0.6898
Low-density lipoprotein (mmol/L)	2.789 ± 0.5427	2.538 ± 0.6474	0.0559
Smoker (*n*, %)	6 (14.29)	8 (18.18)	0.6295
Alcohol consumption (*n*, %)	8 (19.05)	12 (27.27)	0.3727
Hypertension (*n*, %)	7 (16.67)	9 (20.45)	0.6564
Diabetes (*n*, %)	5 (11.9)	6 (13.64)	0.8128
Hyperlipidemia (*n*, %)	22 (52.38)	26 (59.09)	0.5367
Calcium antagonist (*n*, %)	5 (11.9)	7 (15.91)	0.5973
ARB/ACEI (*n*, %)	28 (66.67)	31 (70.45)	0.7092
*β*-blocker (*n*, %)	29 (69.05)	29 (65.91)	0.7596
Nitrate ester (*n*, %)	7 (16.67)	9 (20.45)	0.6564
Statins (*n*, %)	39 (92.86)	42 (95.45)	0.6119
Antiplatelet drug (*n*, %)	42 (100.0)	44 (100.0)	ND
miRNA-22	0.97 ± 0.06	3.28 ± 0.29	<0.001

BMI, body mass index; ARB/ACEI, angiotensin receptor blocker/angiotensin-converting enzyme inhibitor (continuous variables with normal distribution were expressed as mean ± SD).

**Table 2:** TIMI blood flow evaluation of the two groups.

	Control group (*n* = 42)	Coronary slow flow group (*n* = 44)	*P* value
CTFC of LAD	30.83 ± 6.836	20.73 ± 4.406	<0.001
TFC of LCX	28.88 ± 6.041	20.20 ± 3.137	<0.001
TFC of RCA	33.12 ± 5.878	22.09 ± 3.820	<0.001
Mean TFC	30.94 ± 4.044	21.01 ± 2.525	<0.001

TIMI, thrombolysis in myocardial infarction coronary angiography imaging evaluation of coronary artery perfusion; CTFC, corrected TIMI frame count; LAD, left anterior descending artery; LCX, left circumflex artery; RCA, right coronary artery; mean TFC, mean TIMI frame count (continuous variables with normal distribution were expressed as mean ± SD).

## Data Availability

The data used to support the findings of this study are available from the corresponding author upon request.
